# Implementation of sonication and feedback control strategies for targeted hyperthermia in prostate with a commercial MR-guided endorectal ultrasound ablation array

**DOI:** 10.1186/2050-5736-3-S1-O62

**Published:** 2015-06-30

**Authors:** Vasant Salgaonkar, Eugene Ozhinsky, Viola Rieke, Punit Prakash, I-Chow Hsu, John Kurhanewicz, Chris Diederich

**Affiliations:** 1 University of California at San Francisco, San Francisco, California, United States

## Background/introduction

This study presents the implementation of protracted mild hyperthermia (HT) delivery to large contiguous volumes in prostate quadrants or hemi-gland targets by utilizing a commercially available MR-guided endorectal ultrasound (ERUS) phased array designed for thermal ablation (ExAblate 2100, InSightec. Ltd). HT-specific beamforming, sonication and control strategies were devised for the hardware/software limitations imposed with this commercial system.

## Methods

**Figure 1:**
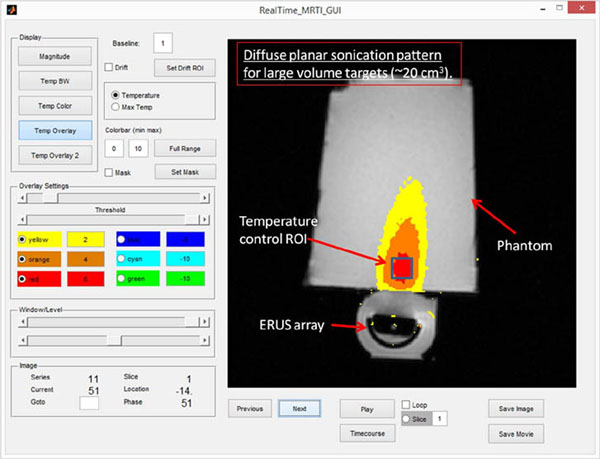
Custom software for real time MR thermometry during hyperthermia

Various array beamforming alternatives and feedback control methods were explored through extensive numerical simulations consisting of patient-specific biothermal modeling for heating with the ExAblate prostate array (2.3 MHz, ~1000 channels), consistent with system constraints such as power densities, sonication durations and switching speeds. Finite element method solvers (Comsol Multiphysics) were utilized to compute 3D thermal distributions for representative patient-specific geometries rendered from serial MRIs (Mimics, 3-Matic). The beamforming, sonication and control parameters ascertained during the simulation studies were implemented on the ExAblate prostate array through animal tissue and phantom experiments under MR temperature imaging (MRTI) [3T, PRFS] performed in real-time with a custom software developed in Matlab for temperature reconstruction (Fig. 1).

## Results and conclusions

**Figure 2:**
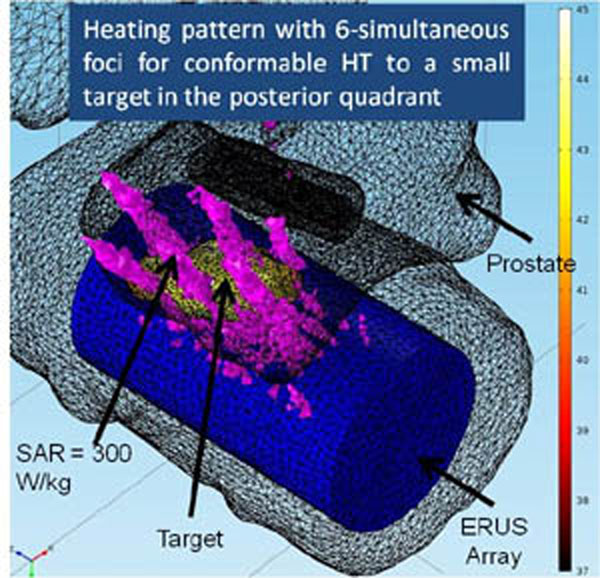
Patient-specific 3D model

The simulation study confirmed conformable HT delivery to focal cancer volumes in a single prostate quadrant using focused heating patterns (simultaneous multi-focus [example in Fig. 2], or curvilinear) and hemi-gland HT using diffused patterns (planar or diverging). 4 oC temperature rises were calculated in 13–23 cm3 volumes for planar or diverging beam patterns at 0.9–1.2 W/cm2, in 1.5-4 cm3 volumes for simultaneous multi-point focus patterns at 2 – 3.4 W/cm2, and in ~6.0 cm3 for curvilinear patterns at 0.75 W/cm2.

**Figure 3:**
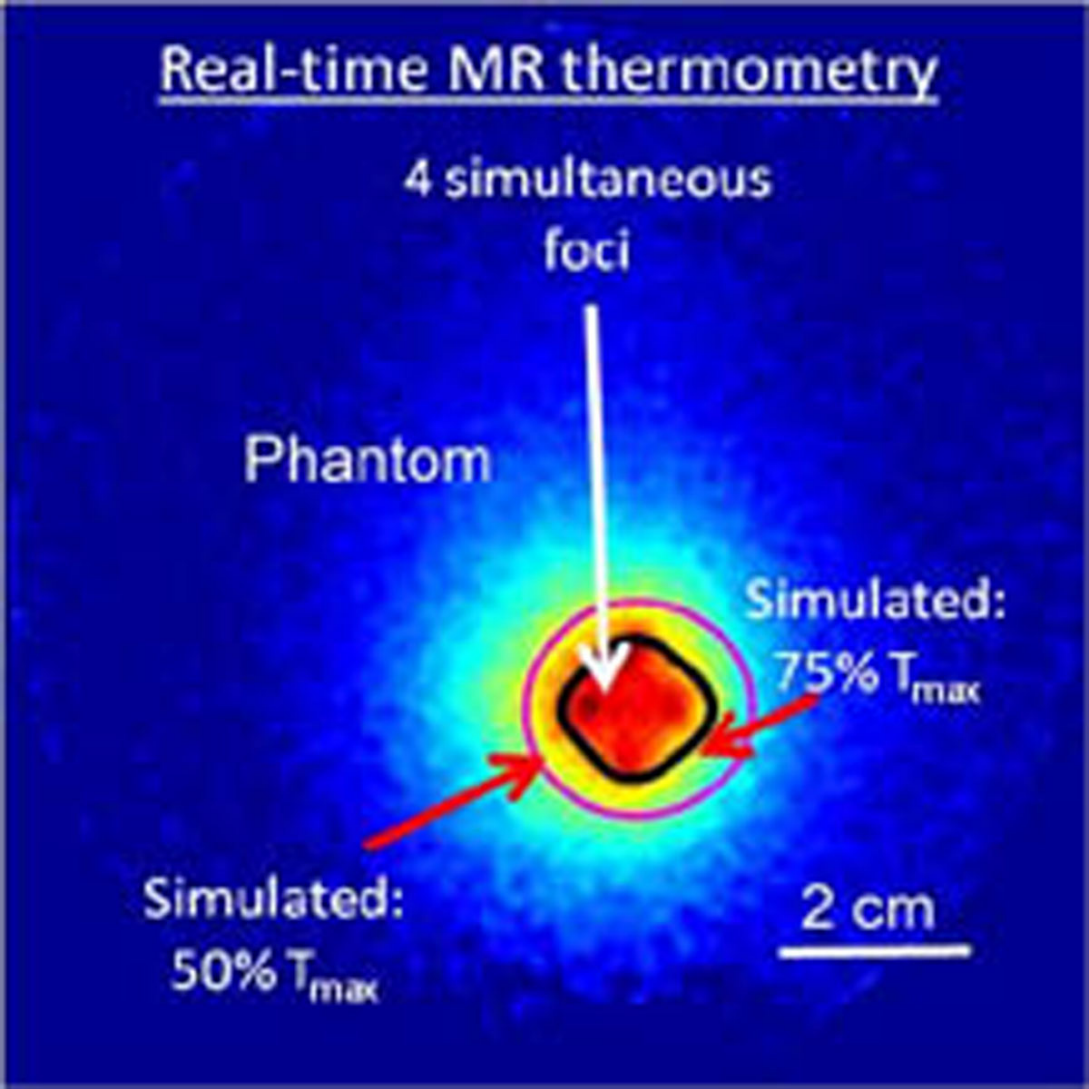
Example of MRTI-based feedback control

**Figure 4:**
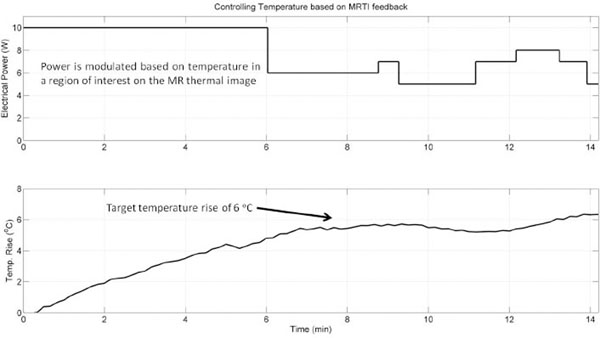
Comparison between modeling and experiment

HT-specific sonications were successfully performed using the ExAblate prostate array and therapeutically relevant temperature rises (4 – 8 oC) were sustained for long-durations (>15 min) in large contiguous volumes. Consistent heat shapes were observed between simulations and tissue phantom experiments for various beamforming schema (example in Fig. 3). MRTI feedback was utilized to control temperature within a target volume through binary, proportional and proportional-integral (PI) control schemes employing temperature-based modulation of sonication power (Fig. 4). Feasibility, practicability and capabilities of the ExAblate ERUS array were evaluated for long-duration, large-volume, temperature-controlled HT through real-time MRTI with the purpose to facilitate rapid clinical translation of targeted prostate HT with the ExAblate prostate array as an adjuvant to focal radiotherapy and targeted chemotherapy.

